# Implementation and outcomes of guideline revisions for the prevention of mother-to-child HIV transmission in Mother Support Programme, Addis Ababa, Ethiopia

**DOI:** 10.1371/journal.pone.0198438

**Published:** 2018-06-21

**Authors:** Alemnesh H. Mirkuzie

**Affiliations:** Ethiopian Public Health Institute, National Data Management Center, Addis Ababa, Ethiopia; Lewis Katz School of Medicine at Temple University, UNITED STATES

## Abstract

About 40% of the new HIV infections in Ethiopia are among children < 15 years of age. The great majority of these infections occur through Mother-to-child HIV transmission (MTCT). For prevention of MTCT, the national guidelines has been revised to incorporate scientific advances in HIV prevention, treatment and care. Since 2005, the country has been implementing a peer mentor programme called Mother Support Group (MSG), which provides psychosocial and adherence support for HIV positive mothers. This study examined implementation of PMTCT guidelines revisions and outcomes of HIV exposed babies in the MSG in Addis Ababa. Retrospective routine data were collected between 2005 and August 2013 from seven randomly selected primary health facilities. Odds ratios and 95% confidence intervals were calculated using logistic regression models. Several guidelines revisions were made between 2001 and 2013 in HIV testing approaches, prophylactic antiretroviral options, infant feeding recommendations and infant HIV testing algorithms. Revisions on the CD4 thresholds were associated with a significant increase in the proportion of women initiating antiretroviral treatment from 0 in 2005 to 62% in 2013. Revisions in infant feeding recommendations led to a 92.3% reported practice of exclusive breastfeeding in 2013 compared to 60.9% in 2005. Two and four percent of the HIV exposed babies were HIV positive by six and 18 months respectively. Not receiving prophylactic ART and receiving mixed feeding were independent predictors for babies having an HIV positive antibody test at 18 months. The rate of HIV status disclosure increased significantly year by year. Over the years, the PMTCT recommendations have moved from having a solo focus on PMTCT to holistic and inclusive approaches emphasizing survival beyond HIV prevention. The data reflect favourable outcomes of HIV exposed babies in terms of averted MTCT though serious gaps in data quality remain. For successful implementation of Option-B plus, the identified gaps in the MSG need to be addressed.

## Introduction

Ethiopia has reported a steady decline in HIV incidence and AIDS related mortality since 2005. The country has also achieved the first target of the Millennium development goal 6, which is halting and reversing the spread of HIV/AIDS [1, 2]. Concerted efforts by the government, local and international partners including local communities has contributed to this progress. Despite the overall significant decline in the HIV incidence, the MTCT remains a significant challenge in controlling the epidemic. Of the 21,000 estimated new HIV infections which occurred in 2013, 40% were among children <15 years old [2].

In 2009, the UNAIDS set a Global plan for the elimination of new HIV infections among children and keeping their mothers alive. The plan prioritized 22 high HIV burden low resource countries in Africa and Ethiopia is one of them [3]. In line with this initiative, at the national level Ethiopia has renewed its commitment and galvanized efforts to eliminate new HIV infections among children by 2015 and to keep HIV positive mothers alive. For this the 2011 revised national PMTCT guidelines introduced a more efficacious ART prophylactic option also called Option A. In option A, HIV positive women having CD4 cell count ≤ 350 cells/mm^3^ are eligible for lifelong ART for PMTCT and for their own health, while triple prophylaxis is prescribed for those who are not eligible for ART [3]. Following its implementation, the country recorded commendable progress. In the 2014 UNAIDS report, the proportion of women accessing prophylaxis antiretroviral therapy (ART) during pregnancy and breastfeeding increased from 9% in 2005 to 55% in 2013 [2]. However, this improvement far from achieving the 90% planned reduction in new HIV infection among children. Later, in 2013 Ethiopia launched Option-B plus to further accelerate progress in PMTCT as well as to improve access to ART for all HIV positive pregnant women for their own health. In Option-B plus, all HIV positive pregnant women receive lifelong ART [3].

Since the introduction of the first national PMTCT guidelines in 2001, Ethiopia has made series of guidelines revisions in 2007, 2011 and 2013 to incorporate new knowledge, global recommendations and scientific advances [4–6]. To facilitate implementation of the national guidelines and PMTCT programme, a Mother Support Group (MSG) has been implemented since 2005 [7, 8]. The MSG is a peer mentor programme inspired by the South African ‘mothers2mothers’ initiative [7]. The MSG intends to increase uptake and adherence to PMTCT services, to facilitate efforts of coping with the virus and to reduce stigma and discrimination among HIV positive pregnant women. IntraHealth, an international non-governmental organization with funding from UNAIDS first introduced and ran the MSG programme in public health facilities until 2008 [7]. Over the years, several NGOs implemented the programme with little government involvement.

Following the inception of the Option-B plus initiative, the federal Ministry of Health took over the MSG programme in 2013. The Option-B plus implementation care model in Ethiopia is to contain all the maternal and exposed babies’ care in the Maternal, Newborn and Child Health (MNCH) care units [9]. The care model promises a one-stop shop approach whereby mother-baby pairs are monitored and followed up through the MTCT at-risk periods in the antenatal clinic. To reduce the added workload on the antenatal clinic that accrues with the implementation of Option-B plus, the MNCH fully integrates the MSG for task sharing. This approach capitalizes on documented successes that “task-sharing” and “task-shifting” have brought in rolling out ART programmes in many resource poor settings including Ethiopia [10, 11]. Following HIV positive diagnosis in antenatal clinic and before ART initiation, women are referred to MSG. In the MSG, mentor mothers provide adherence and psychosocial support, ensure regular follow up of women and their babies and trace defaulters. If babies received an HIV positive diagnosis, the mother-baby pairs will be discharged from the antenatal clinic and referred to ART clinic where the rest of their follow-ups take place.

The MSG programme has been implemented across the country for over a decade but little has been known about its contribution in supporting the national PMTCT programme. According to a 2009 UNAIDS project evaluation, although the MSG programme had a positive significant impact on HIV positive women and their babies, attrition and poor data quality were threatening its success [7]. Nonetheless, the government currently recognises the MSG as an ideal platform for the implementation of Option-B plus. Therefore, the present study document trends and lessons learned using nine years of routine retrospective data from MSG programmes in Addis Ababa. The specific objectives of the study were 1). to assess how the MSG programme handled PMTCT guidelines revisions in the past 2). to assess outcomes of mothers and HIV exposed babies in the programme not only to learn from past experiences but also to have a baseline for monitoring future progress 3) to estimate the rate of HIV infection among HIV exposed infants and 4) to assess data quality.

## Methods

### Study settings

Addis Ababa, the capital of Ethiopia has the highest HIV prevalence in the country. Although the HIV incidence has been steadily declining since 2005, still approximately 5% pregnant women in the city are living with the virus [12]. For the prevention of MTCT, a free PMTCT programme was launched in 2003. In 2004, 24 primary care facilities, referred in this study as health centers started implementing the programme. Following progress in the rolling out of the PMTCT programme more and more health facilities have started implementing every year. The programme has been implemented in accordance with the national PMTCT guidelines revisions and updates that follow scientific advances and global recommendations.

### Evolution of the national PMTCT guidelines

The first national guideline recommended voluntary HIV counselling and testing to identify and enrol HIV positive women in PMTCT programme. Single dose nevirapine (NVP) prophylaxis at birth, exclusive formula as first line infant feeding choice and antibody testing of HIV exposed babies at 18 months of age were among the recommendations. This guidelines has supported the implementation of the national PMTCT programme between 2005 and 2007 (Table 1).

**Table 1:** Chronology of the national PMTCT guideline revisions and recommended interventions in Ethiopia.

Guideline	Recommended prophylaxis	Infant feeding recommendation
**1st developed in 2001**	***Women***	Preferred feeding- Exclusive formula
	Single dose NVP at birth	
	***Baby***	Alternative feeding—Exclusive breast feeding and abrupt cessation at six months
	Single dose NVP within 72 after birth	
**1st revision in 2007**	***Woman***	Preferred feeding—Exclusive breast feeding for the first six months + Complementary feeding from 6 to 18 months
	Pregnancy: ZDV from 28 weeks	
	At birth: ZDV+ NVP + 3T	
	Postpartum: ZDV+ 3TC twice per day for seven days	
	***Baby***	
	At birth: ZDV+ NVP	Alternative feeding—Replacement feeding if AFASS
	Postpartum: ZDV bid for seven days	
**2nd revision in 2011 Option A**	***Woman***	Preferred feeding—Exclusive breast feeding for the first 6 months and complementary feeding from 6 to 12 months
	Pregnancy: ZDV from 14 weeks	
	At birth: NVP + 3TC/ZDV	
	Postpartum: 3TC/ZDV twice per day for seven days	
	***Baby***	Alternative feeding—Replacement feeding if AFASS
	NVP daily from birth through one week after cessation of breast feeding	
	NVP at birth + AZT twice per day for six weeks for infants on replacement feeding	
**3rd revision in 2013 Option B- plus**	***Woman***	No change in infant feeding recommendation
	HAART irrespective of the CD4 cell count and gestational age	
	***Baby***	
	Daily NVP or AZT from birth to four to six weeks of age regardless of infant feeding method	

AFASS—affordable, feasible, acceptable sustainable and safe, ART- antiretroviral therapy, HAART—highly active antiretroviral treatment, NVP—Nevirapine, ZDV—Zidovudine, 3TC- Lamuvudine

A comprehensive revision of the guideline was made in 2007 that shifted the HIV testing approach from opt-in to opt-out. This has made antenatal HIV testing a routine standard of care [4]. The prophylaxis regimen has shifted from mono-therapy to multidrug therapy. This shift was in line with the 2004 WHO recommendations on antiretroviral drug for treating pregnant women and preventing HIV infection in infants [13]. Prophylaxis ART was initiated at 28 weeks of gestation in women with CD4 count ≥ 200 cells/mm^2^, while those who have a CD4 cell count < 200 cells/mm^3^ initiate HAART. This guideline promoted exclusive breastfeeding as the preferred infant feeding method for the first six months, then follows complementary feeding until 18 months of age. The revision also incorporated Deoxyribonucleic acid (DNA) Polymerase chain reaction (PCR) infant HIV testing at six weeks from dried blood sample (DBS) followed by confirmatory HIV antibody testing at 18 months or one week following complete cessation of breastfeeding. The 2007 versions guided the national PMTCT programme implementation from 2008 to 2010 (Table 1).

The 2011 revision was mainly on prophylaxis regimen in accordance with the WHO recommendations made in 2010 that introduced Option-A [5, 14, 15]. In Option-A, prophylaxis ART is initiated in early pregnancy and to be continued until cessation of breastfeeding. Pregnant women whose CD4 count is ≥ 350 cells/mm^3^ receive prophylaxis. Pregnant women whose count < 350 cells/mm^3^ irrespective of clinical stage and those in the WHO stage 3 and 4 initiate HAART (Table 1). The 2011 revision had guided the national PMTCT programme from 2011 to August 2013.

In February 2013, Ethiopia officially launched Option-B plus to accelerate progress to achieve elimination of MTCT. In Option-B plus, women start lifelong ART from the time of HIV diagnosis irrespective of their CD4 cell count, viral load or WHO clinical stage. Single pill fixed dose combination of Tenofovirdisoproxilfumarate, lamuvididne and efavirenz (TDF/3TC/EFV) is the drug of choice. Option-B plus has high efficacy in reducing MTCT and horizontal transmission to sexual partners, potential to increase ART coverage, significant maternal health benefit and has high programme acceptability [2, 9]. Although, Ethiopia’s motivation to move from Option A to Option B plus was primarily to achieve its ambitious goal of eliminating MTCT by 2015, being a country with a generalized epidemic, the programmatic and operational advantages of it were also additional driving forces [9]. Since August 2013 the Option-B plus has been rolled out in public health facilities by replacing all the existing prophylactic ART options [9].

Partner involvement in PMTCT programme and HIV serostatus disclosure to partner have been an integral part of all the PMTCT guidelines in Ethiopia [4–6]. Several studies have shown improved adherence to PMTCT services among women whose partners were involved in the PMTCT programme and those who disclosed their HIV positive status to their partners [16–18]. Moreover, all the guidelines have emphasised the need for linking HIV positive women to treatment, care and support services.

### MSG programme implementation

To optimize the effectiveness of the PMTCT programme, a MSG was launched in 2005 in public health facilities [8]. This peer mentor programme follows women who found to be HIV positive during their peri-partum visits in public health facilities. Only women who wish to participate in the programme are enrolled. The programme offers regular meetings and discussion sessions for the women to express their concerns, pose questions and clarify doubts and myths. Scheduled meetings are often accompanied by a traditional “coffee ceremony” where all women come together to share each other’s experience and to discuss psychosocial and medical issues.

Mentor mothers provide peer counselling on:

- ART (prophylaxis) uptake, possible side effects and the need for strict adherence to ART regimen using their life experiences as example
- Regular attendance for virological and immunological monitoring
- Different infant feeding options and the need for strict adhere to a chosen option
- How to establish and maintain the correct breast feeding practice tailored according to child age
- Partner involvement in PMTCT programme
- HIV positive status disclosure to the partner, suggest strategies on how to disclose and help women to do so
- Safe delivery practices and postnatal care
- Family planning and safe sexual practices (strong emphasis on consistent condom use)
- Cotrimoxazol prophylaxis for women and babies
- HIV testing for exposed babies and treatment initiation for positive babies
- Regular follow up visits for women and their babies.

Moreover, the MSG providers do routine registration; link HIV positive women to appropriate services such as ART, family planning, nutrition programmes, social support and infant HIV testing services. They also dispense prophylactic cotrimoxazol for HIV exposed babies and condoms in some of the MSG sites. The MSG providers are volunteers who only receive transport allowances. The number of MSG providers in each site ranges from three to four.

The MSG initiative receives support from international and local partners involved in the implementation of the PMTCT programme in the country. In the early years, IntraHealth is an international NGO used to provide consistent yet modest technical, financial and logistic supports for the coffee ceremonies, training and transportation allowance for mentor mothers [7]. Later, when Management Science for Health and John Hopkins University took over the programmes, such incentives were reduced [8]. Considering its significant contributions, the national PMTCT guidelines have incorporated the MSG activities since 2007. However, there was no direct support from the government for the programme until the launching of the Option-B plus in 2013. For the successful rollout of the Option-B plus initiative, currently the Federal HIV/AIDS prevention and control office and the Federal Ministry of health have been working jointly to fully integrate the MSG endeavour in the national PMTCT programme.

### Study design

This study, conducted in September 2013 had collected routine retrospective data from 2005 to August 2013 from health centers. Ten public primary care facilities were randomly selected one from each sub-city of Addis Ababa. Of these, three of the health centers were found not to have the MSG programme and hence excluded. The study also excluded hospitals, as they did not have the MSG programme.

#### Ethics approval and consent to participate

The study was ethically approved as part of a project conducted in Addis Ababa in collaboration with the Addis Ababa City Administration Health Bureau and the Addis Ababa University from 2013 to 2015. The project received Ethical approvals from the Addis Ababa City Administration, Health Bureau ethics committee and from the regional ethics committee in Western Norway. For this study, retrospective routine data was collected anonymously from logbooks using client medical registration number as unique identifier. To access the MSG logbooks; study permit was first obtained first from the Addis Ababa City Administration, Health Bureau, then from respective sub-city health bureau’s and from each health center.

### Data collection

The principal investigator, who has been working closely with the Addis Ababa City Administration, Health Bureau and Addis Ababa University since 2009, collected all the data. In each of the selected health facilities, the MSG programme logbooks used for recording routine PMTCT data were reviewed. In the early phase of the programme, there were two major logbooks; one for recording data during pregnancy and the other for recording data after birth. The two logbooks have the mother record number as a unique code. This study used these codes to link the two logbooks. In recent years, the entries in the two logbooks were merged together with the most recent logbook recording both pre-partum and post-partum information including infant HIV testing. There were inconsistencies in the recording and reporting across the MSG sites. This was partly due to different stakeholders running the programmes at different times. Gaps in the quality of data were commonplace, and some sites were worse than others. Despite the changes in PMTCT interventions across the years, there were limited updates in the MSG logbooks.

### Quality of data and ways of handling potential bias due to missing data

The aforementioned factors also presented challenges to ensure data quality and might have introduced bias to the study. In total, there were 1,084 women registered in the MSG logbooks in the seven health facilities from 2005 to August 2013. Of these, 321 (42%) of the data did not have complete information. Missing data is a common problem in studies using routine data collected for reporting purposes. Missing data not only introduce bias but also compromises the statistical power of a study [19–21]. It is more problematic when the missing data is related to an outcome or exposure. In the present study, the missing data appeared to be related to practical and logistic issues within the MSG programme than attrition and study outcomes. Low literacy of the mentor mothers in recording observations and their poor awareness about its significance, inconsistencies in the logbooks, poor monitoring and evaluation of the programme, limited technical and logistic supports to the programme and the challenges surrounding DBS testing (eg. drawing blood sample, taking sample to central laboratory, collecting test results, recording results on logbooks and communicating test results to mothers) had contribution for the poor quality of the MSG reports and for the missing data. To minimize bias due to missing data and to yield least biased estimates, studies use different missing data analysis methods including deletion also called conventional, nonstochastic imputation and stochastic imputation methods [19, 20, 21]. The present study has employed the conventional approach, assuming that the missing data have occurred at random. The study presented only cases with complete data on exposed babies HIV testing outcomes [19, 20].

In a review, Kang indicates, “missing at random does not mean the missing data can be ignored” [19]. In this regard, the present study undertook sensitivity analysis in addition, to account for potential bias due to the missing data. [22].

### Data analyses

Data were analysed using the SPSS statistical package version 21. The number and percentages of women and babies who received prophylaxis in relation to PMTCT guidelines revisions, trend in infant feeding practices in relation to PMTCT guidelines revisions, percentage of babies having DNA PCR and HIV antibody testing, number and percentage of women who disclosed their HIV status to partner and partner HIV test results were calculated. Using bivariate and multivariate logistic regression analyses, odds ratio (OR) and 95% confidence interval (CI) were calculated to determine predictors for exposed infant HIV test status. The predictor variables entered in the bivariate model were year enrolled in MSG, women ART medicine, infant prophylaxis, infant feeding methods, partner HIV test status and disclosure to partner. Of these, infant prophylaxis, infant feeding methods and partner HIV test status showed a chi square statistical p-value ≤ 0.2 and hence entered into the multivariables model to control for potential confounding. A variable having CIs including one for adjusted OR was not considered as an independent predictor. Sensitivity analysis was done to account for potential bias due to missing data as described above.

## Results

In the MSG programme, 764 records with complete data on HIV test results for exposed babies were retrieved between 2005 and 2013. Of these, 76 (9.9%) were between 2005 and 2007 when the 2001 (first) PMTCT guideline was implemented, 219 (32.6%) were between 2008 and 2011 when the 2007 revised PMTCT guideline was implemented and 444 (57.3%) were recorded between 2011 and 2013 when the 2011 PMTCT guideline was implemented (Table 2).

**Table 2:** Characteristics of women and HIV exposed babies enrolled in MSG programmes from 2005 to 2013, Addis Ababa.

Variable	Number (%)
***Year enrolled in MSG/PMTCT***	
2005 to 2007	76 (9.9)
2008 to 2010	219 (32.6)
2011 to 2013	436 (57.5)
***Delivery place***	
Health facility	748 (97.9)
Home	16 (2.1)
***Infant prophylaxis***	
Yes	736 (96.3)
No	28 (3.7)
***Women ART status***	
Prophylaxis	407 (53.3)
HAART	353 (46.3)
No	4 (0.5)
***Infant feeding methods***	
Exclusive breast-feeding	691 (89.9)
Exclusive formula	63 (8.2)
Mixed feeding	10 (1.8)
***Disclsure to partner***	
Yes	421 (55.1)
No	90 (11.8)
Unknown	245 (32.5)
Partner dead	5 (0.7)
***Partner HIV test status***	
Negative	136 (17.8)
Positive	243 (31.8)
Unknown	380 (49.7)
Partner dead	5 (0.7)
Variable	Number (%)
Year enrolled in MSG/PMTCT	
2005 to 2007	76 (9.9)
2008 to 2010	219 (32.6)
2011 to 2013	436 (57.5)
Delivery place	
Health facility	748 (97.9)
Home	16 (2.1)
Infant prophylaxis	
Yes	736 (96.3)
No	28 (3.7)
Women ART status	
Prophylaxis	407 (53.3)
HAART	353 (46.3)
No	4 (0.5)
Infant feeding methods	
Exclusive breast-feeding	691 (89.9)
Exclusive formula	63 (8.2)
Mixed feeding	10 (1.8)
Disclosure to partner	
Yes	421 (55.1)
No	90 (11.8)
Unknown	245 (32.5)
Partner dead	5 (0.7)
Partner HIV test status	
Negative	136 (17.8)
Postive	243 (31.8)
Unknown	380 (49.7)
Partner dead	5 (0.7)

**Fig 1:**
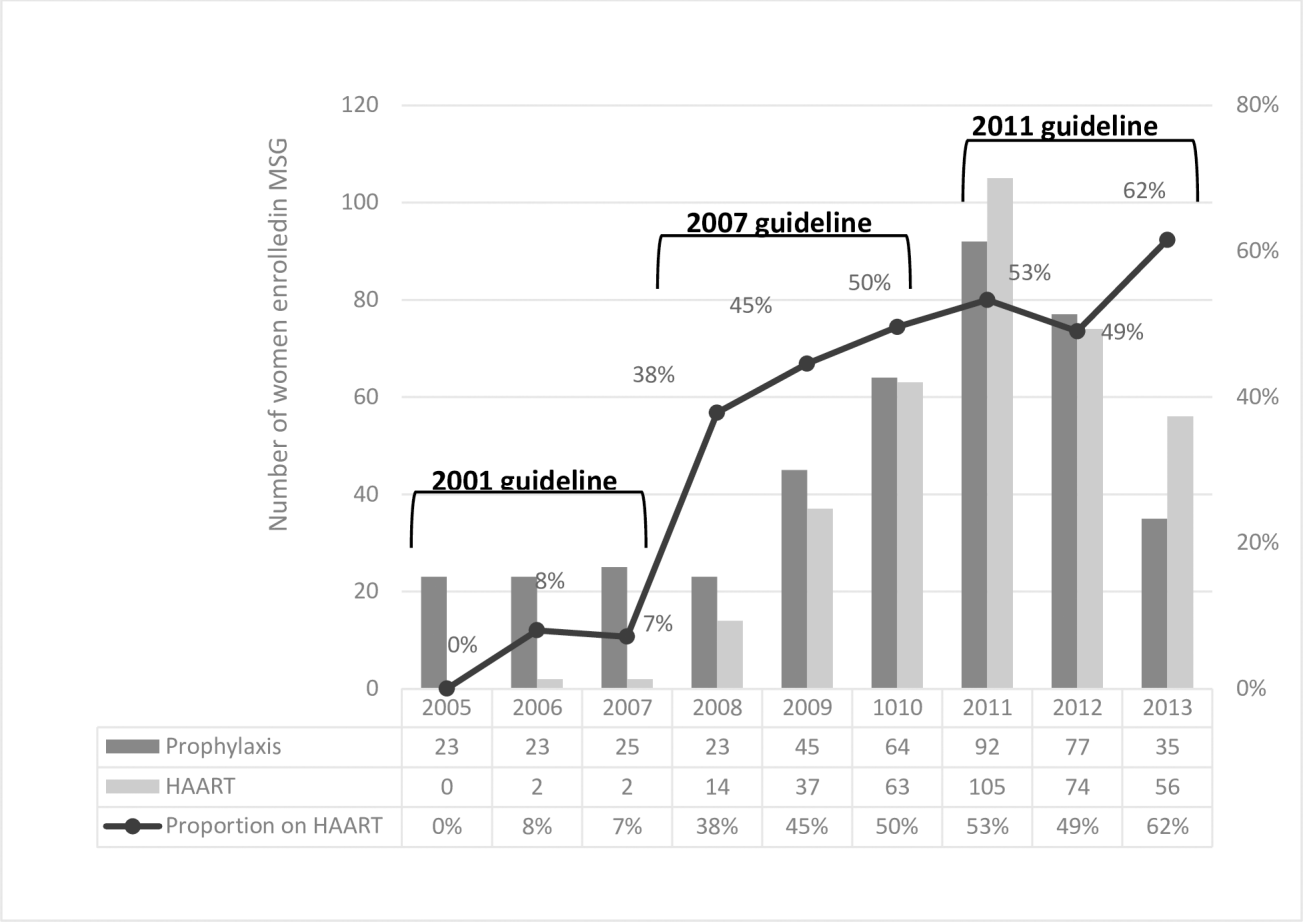
Trend data showing proportions of women receiving HAART over the nine years in the MSG programme, Addis Ababa.

The great majority, i.e. 748 (97.9%) of the babies gave birth at health facilities (Table 2). Five women (0.9%) did not take any ART prophylaxis/HAART, while 353 (46.3%) were on HAART. In parallel with the increased CD4 thresholds to initiate HAART, the number and proportions of women receiving HAART showed an increasing trend from 0 in 2005 to 62% in 2013. A dramatic increase in the proportion of women receiving HAART occurred between 2007 and 2008 from 7% to 38% (Fig 1).

**Fig 2:**
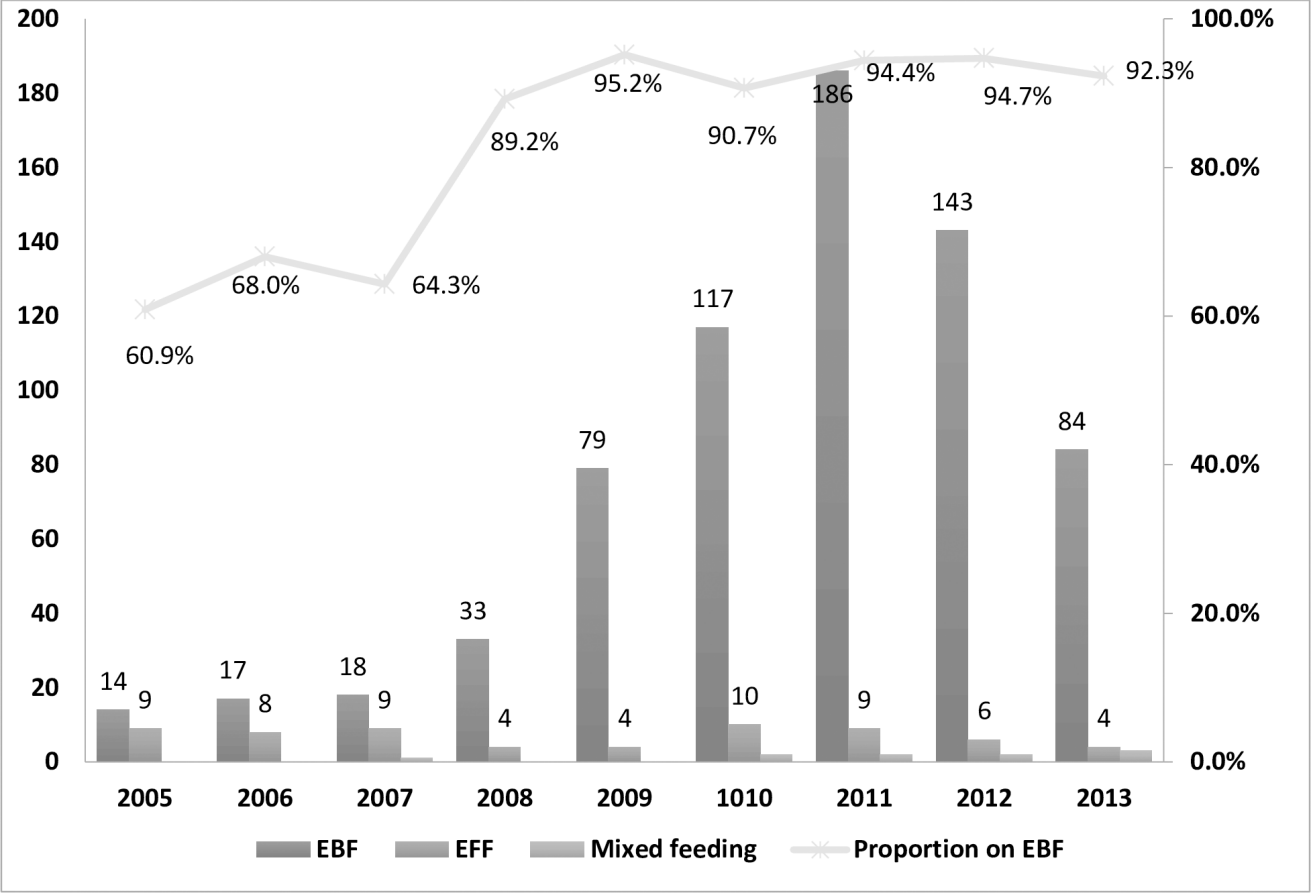
Patterns of infant feeding practices from 2005 to 2013 among babies with DNA PCR/HIV antibody tests in the MSG programme, Addis Ababa. NB: EBF stands for exclusive breast-feeding; EFF stands for exclusive formula feeding.

Of the 764 babies included in this study, 691 (89.9%) were reported to have received exclusive breastfeeding while only 10 (1.8%) received mixed feeding. Over the years, the proportions of babies reportedly receiving exclusive breastfeeding showed an increasing trend from 60.9% in 2005 to 92.3% in 2013. During the implementation of the 2001 guideline, less than 70% of the babies reportedly received exclusive breastfeeding. Following the changes in infant feeding recommendation from 2007, the proportions of babies receiving exclusive breastfeeding had increased significantly (Fig 2).

From the sample, five women reported to have lost their partner to HIV and 421 (55.1%) had disclosed their HIV positive status to their partners. The proportion of women whose HIV status disclosure was not known had shown a decreasing trend from 56.6% during the implementation of the 2001 PMTCT guideline to 31.1% during the implementation of the 2011 PMTCT guideline. Among the 516 women whose HIV disclosure status was known, 426 (82.6%) had reported that they disclosed their HIV positive status to their partners. In total, 384 partners HIV status was recorded of which 136 (35.4%) had HIV discordant results (Table C in S1 File, Table 3).

**Table 3:** Predictors of HIV positive antibody test among HIV exposed babies followed in MSG programmes in Addis Ababa from 2005 to 2013.

*Variable*	*Rapid antibody test result*		*Bi-variable analysis*	*Multi-variable analysis*
	*Negative n = 49 (%)*	*Positive n = 22 (%)*	*OR(CI)*	*AOR (CI)*
***Year enrolled in MSG***				
***2011 to 2013***	*261 (96.7)*	*9 (3.3)*	*1*	
***2008 to 2010***	*167 (94.9)*	*9 (5.1)*	*1.63 (0.49, 5.46)*	
***2005 to 2007***	*71 (94.7)*	*4 (5.3)*	*1.56 (0.61, 4.02)*	
***Infant prophylaxis***				
***Yes***	*483 (96.2)*	*19 (3.8)*	*1*	*1*
***No***	*16 (84.2)*	*3 (15.8)*	*4.77 (1.28, 17.76)*	*5.50 (1.38, 21.95)*
***Women ART medicine***				
***Prophylaxis***	*218 (95.6)*	*10 (4.4)*	*1*	
*HAART*	*279 (95.9)*	*12 (4.1)*	*0.94 (0.40, 2.21)*	
***Infant feeding methods***				
***EBF***	*445 (96.7)*	*15 (3.3)*	*1*	*1*
***EFF***	*49 (92.5)*	*4 (7.5)*	*2.42 (0.77, 7.58)*	*2.74 (0.84, 8.88)*
***Mixed feeding***	*5 (62.5)*	*3 (37.5)*	*17.80 (3.89, 81.47)*	*16.43 (3.39, 79.54)*
***Partner HIV test status***				
***Negative***	*176 (97.2)*	*5 (2.8)*	*1*	*1*
***Postive***	*101 (98.1)*	*2 (1.9)*	*1.44 (0.27, 7.53)*	*1.31 (0.25, 6.93)*
***Unknown***	*222 (93.7)*	*15 (6.3)*	*3.41 (0.77, 15.20)*	*2.35 (0.51,10.85)*
***Disclosure to partner***				
***Yes***	*306 (96.5)*	*11 (3.5)*	*1*	
***No***	*49 (96.1)*	*2 (3.9)*	*0.58 (0.23, 1.42)*	
***Unknown***	*144 (94.1)*	*9 (5.9)*	*0.65 (0.14, 3.13)*	

*NB- CI stands for Confidence Interval*, *OR stands for Odds Ratio*, *AOR stands for adjusted Odds Ratio*

Of the 764 babies with complete data on HIV test result, 640 had a DNA PCR test done from DBS before six months of age and 521 had HIV antibody tests at 18 months or after. Three hundred and ninety seven babies had both DNA PCR and HIV antibody tests. Of which one baby who had an HIV positive DNA PCR test was found HIV negative for HIV antibody test at 18 months. Of the 640 babies who had a DNA PCR test 12 (1.9%) had HIV positive results (Table A in S1 File, Table 1). Among the 521 babies with HIV antibody tests, 22 (4.2%) were HIV positive (Table B in S1 File, Table 2). In bivariate models, the odds of having an HIV antibody positive test result was higher among babies who did not receive prophylaxis (OR 4.77, CI(1.28, 17.76)) than those who received prophylaxis and among babies who received mixed feeding (OR 16.43, CI (3.39, 79.54)) than those who received exclusive breast-feeding. Controlling for potential measured confounders in the multivariable model, babies not receiving prophylaxis (OR 5.50 CI(1.38, 21.95)) and receiving mixed feeding (OR 16.43, CI (3.39, 79.54)) were independent predictors for babies having an HIV antibody test result at 18 months. (Table 3).

## Discussion

The study reported implementation and outcomes of PMTCT guideline revisions in MSG programme. The HIV testing strategy in the 2001 guideline appeared to be restrictive as women were expected to actively opt-in to have HIV testing. Consequently, few women were enrolled in the PMTCT and MSG as shown in Table 2. A dramatic increase in the proportion of women enrolled in the PMTCT and MSG was recorded following the implementation of the 2007 guideline that introduced opt-out HIV testing approach. Consistent with this finding, a former study that used routine retrospective data from the national PMTCT programme reported that the proportion of women who tested for HIV and collected their test results have increased from 51% in 2007 to 84% in 2009 following the promotion of routine antenatal HIV testing in Addis Ababa [16].

PMTCT recommendations made in the early days of the epidemic focused mainly on how to protect exposed babies from getting HIV, with little considerations for maternal health. Fig 1 has shown an increasing trend in the proportion of women receiving HAART over the years, from 0 in 2005 to 62% in 2013, where the major increase happened between 2007 and 2008. Recent studies are in favour of early initiation of ART to achieve high-level viral suppression to lower MTCT and to maximize health benefits to the women [16]. Following the changes in the CD_4_ thresholds for initiating HAART, an increased proportion of women have benefited. Compared to a previous study in Addis Ababa that reported about 33% HAART initiation among pregnant women in 2009, the proportion reported here for the same year was high [23]. These disparities could be attributed to differences in data sources where the present study used data from the MSG programme while the former study compiled data from the national PMTCT programme. Although, the MSG is part of the national PMTCT programme, enrolment to MSG is not compulsory. Therefore, unlike the national PMTCT programme utilized by women from all walks of life, the MSG programme often attracts symptomatic HIV positive women having poor health, low CD_4_ cell counts and those who need psychosocial support.

Early infant feeding recommendations had given more focus on prevention of HIV transmission to HIV exposed babies with little considerations on the survival advantages of the recommended feeding methods. As shown in Fig 2, the proportion of women reporting exclusive breastfeeding had increased significantly over the years. In the 2001 PMTCT guidelines, formula was the preferred infant feeding option as long as it was affordable, feasible, acceptable, sustainable and safe (AFASS). During this period, only 61% of the babies received breastfeeding. The major lessons learned during the implementation of this option in resource-constrained settings was that infants who received formula did not have improved overall survival compared to breast fed infants [15, 24]. When the 2007 guideline recommending exclusive breastfeeding for the first six months as the preferred infant feeding option was implemented, a significant increase in reported exclusive breastfeeding practice were documented. Further increases in reported breastfeeding practices were observed during the implementation of the 2011 guidelines that recommends universal breastfeeding so long as HIV exposed babies are receiving ART prophylaxis throughout the breastfeeding period. This recommendation somewhat normalized the infant feeding practices in the context of HIV. Further increases in breastfeeding practices among HIV positive women could occur following implementation of the Option-B plus regimen that removes any restriction to breast-feeding for women receiving lifelong ART.

Of the infants who had PCR tests before six months of age, 2% were HIV positive. This finding is comparable with clinical trial data demonstrating the effectiveness of multidrug prophylaxis in reducing MTCT [25]. By contrast, the rate reported in the present study was lower than the 8.2% DNA PCR test positive rate reported in a study that compiled a national PMTCT programme report for the same period [16]. Another prospective cohort study from Addis Ababa in 2009 reported 8.4% rate of MTCT at six weeks postpartum [16]. The rate of MTCT from pregnancy to cessation of breastfeeding in the present study was 4.2% among the infants tested for HIV antibody at 18 month. This rate is much lower than the 14% rate of MTCT reported from PMTCT programme data in Addis Ababa [16]. These discrepancies could be attributed to first, large proportions of women in this study had initiated HAART that could have reduced the risk of MTCT. Second, the women in the MSG programme could have improved adherence to prophylaxis/HAART and infant feeding recommendations because of the continuous counselling and support they receive in the programme. Third, it could also be due to biased sample as described in the methods section.

The odds of having HIV positive rapid antibody test results were 15 times higher among babies who received mixed feeding compared to babies who received exclusive breastfeeding. Consistent with our findings, health facility based studies in Gondor and Woliso towns, Ethiopia, reported that babies who received mixed feeding were over four and six times at increased risk of acquiring HIV than those who received exclusive breastfeeding respectively [26, 27]. It is important to interpret the findings of the present study with caution as the study included only those babies whose HIV test results were recorded in the MSG. The lost to follow-ups would have different adherence profiles. Even among the MSG participants’ who were followed up until infant HIV test, those whose babies became HIV positive or died, would be more likely to quit the programme without reporting what has happened. Therefore, the rate of HIV infection among the HIV exposed babies reported here could have been an underestimation of the actual prevalence. In a study conducted in Uganda, the HIV prevalence among babies who completed their follow up were about 50% lower than those who became lost to follow up [22]. Since, the study has used limited samples for estimating the risk of MTCT through infant feeding practices, readers are encouraged to interpret this finding cautiously.

As the study used data from routine programme, gaps in the data quality are expected and might have an effect on the external validity of the study. Consistent with the present study, a UNAIDS evaluation in 2009 reported that among 895 mothers who were enrolled in the MSG programmes across Ethiopia, only 455 (51%) the women and 430 (48%) of their babies received prophylaxis [8]. Reporting of outcomes only for cases having complete follow up might potentially underestimate the risk of MTCT whilst overestimating prophylaxis uptake and adherence by the women and babies and HIV status disclosure to partner. To address this concerns, the study estimated the potential bias introduced due to lost to follow up by using sensitivity analysis. According to a study in Uganda that compared the rate of HIV infection between babies who had follow up and those who became lost to follow up, the rate of HIV infection was twice as high among those who became lost to follow up [22]. Taken this finding into consideration, the present study estimated the rate of HIV for the 42% babies with no HIV antibody test result. Then the 95% confidence intervals for the estimates were checked to assess whether the two samples were from the same population or not. The findings have shown an overlapping confidence interval indicating that the two samples might not be from different populations. The result has given some assurance that babies with missing data on rapid HIV test result might not have a different HIV status by 18 months of age compared to babies having complete data. The study maximized the data quality by employing complete case analyses, which is a conventional method for handling data missing at random, as the missing in this study was primarily due to technical and logistic issues in recording and reporting.

## Conclusions

The study documented implementations of revisions on the national PMTCT guidelines, trends and outcomes of HIV exposed babies who were followed in MSG programme. Recent revisions in infant feeding recommendations that promote breastfeeding for improving child survival appeared to be well adhered. The findings indicate a consistently low rate of MTCT across the different guidelines implementations. However, the findings of the study might be biased due to missing data. The missing data seem to be related to poor data quality and data management at the MSG sites. The quality gaps in the reports could be attributed to low literacy of the mentor mothers, inadequate programme monitoring and evaluation, limited updates in registers and inconsistent training despite guidelines changes, frequent change of NGOs running the programme, high staff turnover and limited state ownership. Hence, in order to use the MSG platform for successful rolling out of the Option-B plus, local and international partners involved in this endeavour could capitalize on the lessons learned and should address identified gaps. These include 1) proper integration of the MSG to MNCH programmes, 2) strengthening state ownership of the programme to ensure sustainability and continuous programme support, 3) regular technical and logistic support as well as up-to-date training for mentor mothers and 4) close monitoring and evaluation of the programme to ensure data quality.

## Supporting information

S1 File Table A: Number and parentage of babies having DBS test and babies having missing data on DBS test results. Table B: Number and parentage of HIV exposed babies having rapid antibody HIV test. Results by 18 months of age and babies with missing data on rapid antibody test results by 18 months of age. Table C: Number and percentage of women whose partner HIV testing status were reported.
(DOCX)

## Abbreviations

AFASS: Affordable, feasible, acceptable, sustainable and safe
ART: Antiretroviral therapy
DBS: Dried blood sample
DNA: Deoxyribonucleic acid
EBF: Exclusive breast feeding
EFF: Exclusive formula feeding
MNCH: Maternal, newborn and child health
MSG: Mother support group
HAART: Highly active antiretroviral treatment
MTCT: Mother to child HIV transmission
NVP: Nevirapine
PCR: Polymerase chain reaction
PMTCT: Prevention of mother to child HIV transmission
UNAIDS: United nations programme for HIV/AIDS
ZDV: Zidovudine
3TC: Lamuvudine
