# Correction: Implementation and outcomes of guideline revisions for the prevention of mother-to-child HIV transmission in Mother Support Programme, Addis Ababa, Ethiopia

**DOI:** 10.1371/journal.pone.0200687

**Published:** 2018-07-10

**Authors:** Alemnesh H. Mirkuzie

An additional affiliation for the author is not indicated. Alemnesh Mirkuzie is also affiliated with Center for International Health, University of Bergen, Bergen, Norway.
