# Supplementary material for: Implementation and outcomes of guideline revisions for the prevention of mother-to-child HIV transmission in Mother Support Programme, Addis Ababa, Ethiopia
Source: PLoS One. 2018 Jun 21;13(6):e0198438. doi: 10.1371/journal.pone.0198438 (PMC6013243; doi:10.1371/journal.pone.0198438)
Supplement: S1 File — Table A: Number and parentage of babies having DBS test and babies having missing data on DBS test results. Table B: Number and parentage of HIV exposed babies having rapid antibody HIV test. Results by 18 months of age and babies with missing data on rapid antibody test results by 18 months of age. Table C: Number and percentage of women whose partner HIV testing status were reported. (DOCX) [file pone.0198438.s001.docx]

**Supporting Information, Table A –C in S1 file :** additional information which are not included in the main manuscript. Table A: Number and parentage of babies having DBS test and babies having missing data on DBS test results, Table B: Number and parentage of HIV exposed babies having rapid antibody HIV test results by 18 months of age and babies with missing data on rapid antibody test results by 18 months of age, Table C: Number and percentage of women whose partner HIV serostatus was reported

Table A

| **Variable** | **Number (%)** |
| --- | --- |
| DBS test negative | 628 (97.2) |
| DBS test positive | 12 (1.8) |
| Total | 640 |
| *Babies with missing data* | **124** |

Table B

| **Variable** | **Number (%)** |
| --- | --- |
| HIV negative | 499 (93.9) |
| HIV positive | 22 (4.1) |
| Total | 521 |
| *Babies with missing data* | ***243*** |

Table C

| **Variable** | **Number (%)** |
| --- | --- |
| HIV negative | 137 (35.4) |
| HIV positive | 249 (63.7) |
| Total | 384 |
| Partner with unknown HIV status | 380 |

|  |
| --- |
